# Bacterial microbiome and host inflammatory gene expression in foreskin tissue

**DOI:** 10.1016/j.heliyon.2023.e22145

**Published:** 2023-11-14

**Authors:** Brandon S. Maust, Stefan Petkov, Carolina Herrera, Colin Feng, Bryan P. Brown, Limakatso Lebina, Daniel Opoka, Andrew Ssemata, Natasha Pillay, Jennifer Serwanga, Portia Seatlholo, Patricia Namubiru, Geoffrey Odoch, Susan Mugaba, Thabiso Seiphetlo, Clive M. Gray, Pontiano Kaleebu, Emily L. Webb, Neil Martinson, Francesca Chiodi, Julie Fox, Heather B. Jaspan, CHAPS team

**Affiliations:** a Center for Global Infectious Disease Research, Seattle Children's Research Institute, Seattle, WA, 98109, USA; b Division of Infectious Disease, Dept of Pediatrics, University of Washington School of Medicine, Seattle, WA, 98195, USA; c Department of Microbiology, Tumor and Cell Biology, Karolinska Institutet, Stockholm, 171 77, Sweden; d Department of Infectious Disease, Faculty of Medicine, Imperial College London, London, W2 1PG, UK; e Perinatal HIV Research Unit, University of the Witwatersrand, Johannesburg, 2000, South Africa; f Medical Research Council, Uganda Virus Research Institute, Entebbe, Uganda; g London School of Hygiene & Tropical Medicine, Uganda Research Unit, Entebbe, Uganda; h Institute of Infectious Disease and Molecular Medicine, University of Cape Town, Cape Town, 7925, South Africa; i Division of Molecular Biology and Human Genetics, Department of Biomedical Sciences, Stellenbosch University, Stellenbosch, 7602, South Africa; j Department of Infectious Disease Epidemiology, London School of Hygiene & Tropical Medicine, London, WC1E 7HT, UK; k Faculty of Life Sciences & Medicine, School of Immunology & Microbial Sciences, Kings College, London, WC2R 2LS, UK

## Abstract

The penile epithelial microbiome remains underexplored. We sequenced human RNA and a segment of the bacterial 16S rRNA gene from the foreskin tissue of 144 adolescents from South Africa and Uganda collected during penile circumcision after receipt of 1–2 doses of placebo, emtricitabine + tenofovir disoproxil fumarate, or emtricitabine + tenofovir alafenamide to investigate the microbiome of foreskin tissue and its potential changes with antiretroviral use. We identified a large number of anaerobic species, including *Corynebacterium acnes,* which was detected more frequently in participants from South Africa than Uganda. Bacterial populations did not differ by treatment received, and no differentially abundant taxa were identified between placebo versus active drug recipients. The relative abundance of specific bacterial taxa was negatively correlated with expression of genes downstream of the innate immune response to bacteria and regulation of inflammation. Our results show no difference in the tissue microbiome of the foreskin with short-course antiretroviral use but that bacterial taxa were largely inversely correlated with inflammatory gene expression, consistent with commensal colonization.

## 1 Introduction

The penile foreskin and its microbiome are important determinants of genital health, but the scientific literature is scant on the typical bacterial constituents and their relationship to tissue inflammation. Previous work using swabs of the coronal sulcus or urethra has shown a predominance of anaerobic species in the microbiota [[1], [2], [3], [4], [5], [6], [7], [8], [9], [10], [11]] and reported associations between species such as *Prevotella* and increased mucosal inflammation and risk of Human Immunodeficiency Virus (HIV) acquisition [4,12]. Following circumcision, the surface microbiota shifts to be dominated by more aerobic species as found on other skin surfaces, which are associated with decreased inflammation and reduced risk of HIV acquisition [6,13,14].

In addition to condom use [15,16], pre-exposure prophylaxis (PrEP, taking antiretroviral medications to prevent HIV infection) [17] is also effective at reducing HIV incidence in men, however the contribution of the penile microbiome to its mechanism has not been fully explored. The most commonly prescribed antiviral regimen for PrEP globally is an oral combination of two nucleoside reverse transcriptase inhibitors: emtricitabine (FTC), a cytidine analog, with one of two forms of tenofovir (TFV), an adenosine analog: tenofovir disoproxil fumarate (TDF) or tenofovir alafenamide (TAF) [18]. Limited data also show a complex relationship of these ARVs with genital bacteria. When applied topically to the vagina, *L. crispatus* was shown to endocytose TFV then either actively metabolize or release it back into the environment [19]. Similarly, *Gardnerella vaginalis* and other anaerobes have been shown to metabolize TFV [20] or block its entry into cells by secretion of adenine [19]. Antiretrovirals may also theoretically alter bacteriophage populations which can dramatically reshape the bacterial component of the microbiome which they infect [21]. At the rectal mucosa, small studies have investigated the effects of oral FTC with TDF on the bacterial microbiome and innate inflammatory pathways in men who have sex with men (MSM) and transwomen [[22], [23], [24], [25]] but with varying results.

Within the CHAPS clinical trial, young men from Uganda and South Africa were randomized to 1 to 2 doses of placebo, FTC with TDF, or FTC with TAF, prior to medical penile circumcision. Foreskin tissue was collected and subjected to both 16S rRNA sequencing and RNASeq to characterize the bacterial microbiome and inflammatory gene expression. Additional studies in this cohort have shown differences in mitochondrial gene expression [26] but no differences in inflammatory gene expression [26] or lymphoid/myeloid cell density in the foreskin [27] between treatment arms. We hypothesized that there would be a relationship between the microbiota and inflammatory gene expression, but that short courses of PrEP, as utilized in a dose-finding trial, would not result in significant disruptions of either.

## 2 Materials and methods

### 2.1 Study participant details

The Combined HIV Adolescent PrEP and Prevention Study (CHAPS) was a randomized controlled trial that enrolled 144 men living without HIV aged 13–24 years between 2019 and 2021 from the Chris Hani Baragwanath Academic Hospital in Soweto, South Africa (n = 72) and the Entebbe Regional Referral Hospital in Entebbe, Uganda (n = 72). Inclusion criteria were male sex at birth, hemoglobin >9 g/dL, weight >35 kg, two successive negative rapid HIV antibody tests, and clinical eligibility for surgical circumcision [28]. Exclusion criteria were conditions precluding circumcision or receipt of the study medications. Participants were randomized to placebo versus FTC with either TDF or TAF for 1–2 days prior to surgical penile circumcision to investigate ARV dosing for on-demand PrEP.

### 2.2 Method details

#### 2.2.1 Study procedures and specimen collection

All participants underwent a physical exam at study entry and completed survey instruments including sexual history at the randomization visit. At the circumcision visit, they provided midstream urine for *Chlamydia trachomatis* (CT) and *Neisseria gonorrhea* (GC) testing via nucleic acid amplification testing (NAAT) prior to surgery. If an asymptomatic sexually transmitted infection was diagnosed, antibiotic treatment was prescribed at the post-operative visit.

Penile circumcision was performed using the dorsal slit method and the removed prepuce was placed immediately in cold Dulbecco's Modified Eagle Medium and shipped on ice within 1 h (median 30 min) to the local laboratories in Uganda (Medical Research Council/Uganda Virus Research Institute) and South Africa (Perinatal HIV Research Unit in Johannesburg) for processing. Smaller, 5–7 mm^2^-sized sections were stored dry at −80 °C until the samples were transported on dry ice to the Seattle Children's Research Institute, U.S.A for microbiome studies and to the Karolinska Institutet, Sweden for transcriptome analyses.

#### 2.2.2 16S rRNA analysis

At the time of analysis, vials were thawed and approximately 25 mg of tissue was dissected and processed by a customized Qiagen PowerSoil Pro protocol [29] for extraction of DNA using a QIAcube instrument. A negative extraction control consisting of solution CD1 without specimen was also included. The specimens from each collection site were extracted on single plates. The resulting total DNA was diluted 1:4 to reduce PCR inhibition.

The *16S* rRNA gene V3–V4 region was amplified using 357F/806R universal primers for 20 cycles of PCR as previously described [30] for each specimen along with a negative PCR control reaction consisting of reaction mix without DNA template for each replicate and evenly and staggered genomic DNA from mock bacterial libraries (BEI Resources) as positive sequencing controls. The amplified products were purified using Agencourt AMPure XP beads (Beckman Coulter) and submitted to an additional 10 rounds of PCR with indexing primers (Illumina). The resulting libraries were pooled by volume with specimens at 100× the positive controls. The resulting library comprising all participants was purified using a MinElute PCR purification column (Qiagen), followed by the QiaQuick gel extraction kit (Qiagen). The cleaned library was quantitated using qPCR (NEBNext Library Quant Kit for Illumina), then pooled with PhiX, denatured, and loaded onto a MiSeq instrument (Illumina) with a v3 2 × 300 flow cell following the manufacturer's protocol.

Sequences were de-multiplexed using Illumina's BaseSpace workflow. Primers and adapters were removed by cutadapt 2.7 [31]. Sequences were further trimmed for quality, then filtered and merged using dada2 1.22.0 [32] to generate amplicon sequence variants (ASVs). Taxa were annotated using the Silva 138.1 database [33] with additional genital-associated species [34] using a 100 % nucleotide identity threshold. The phyloseq 1.40.0 [35] and vegan 2.6-2 [36] R packages were used to create ASV tables and calculate diversity measures. ASV sequences were aligned using ssu-align 0.1.1 [37], and a maximum likelihood phylogeny was generated using PhyML 3.3.20220408 [38] with a GTR substitution model. Contaminating sequences were identified by their presence in negative controls for the extraction and PCR amplification or mock community using decontam 1.16.0 [39] and microfiltR [40]. After decontamination, specimens with fewer than 25-fold as many reads than extraction and PCR controls were excluded. For differential abundance analysis, decontaminated ASVs were filtered with prevalence≥10 % and relative abundance threshold of 1 × 10^−4^ before combining counts for all ASVs classified as the same species. ALDEx2 1.28.1 [41], ANCOM-BC 1.6.2 [42], and DESeq2 1.36.0 [43] (using the poscounts factors estimation) were used for differential abundance testing to overcome the documented limited power and accuracy of these tools when used individually on 16S data sets which contain a high proportion of zero counts [44,45].

#### 2.2.3 RNAseq of foreskin tissue

Details of the RNASeq protocol have been published previously [26]. Briefly, foreskin samples were disrupted and homogenized using a Tissuelyzer (Qiagen) and total RNA isolated using the RNeasy Kit (Qiagen) according to manufacturer's instructions. RNA was subjected to quality control with Agilent Bioanalyzer (Agilent). To construct libraries suitable for Illumina sequencing, the Illumina stranded mRNA prep ligation sample preparation protocol was used with starting concentration of 200 ng total RNA. The protocol includes mRNA isolation, cDNA synthesis, ligation of adapters and amplification of indexed libraries. The yield and quality of the amplified libraries were analyzed using Qubit by (Thermo Fisher) and the Agilent Tapestation (Agilent). The indexed cDNA libraries were normalized and combined, and the pools were sequenced on the Illumina Novaseq 6000 S4 flowcell to generate 150 bp paired-end reads.

Sample demultiplexing was performed using bcl2fastq 2.20.0 (Illumina), and quality and adapter trimming of reads was performed using Cutadapt 2.8 [31]. Sample quality was assessed using FastQC 0.11.8 (Babraham Bioinformatics) and MultiQC 1.7 [46]. Reads were aligned to the Ensembl GRCh38 reference genome using STAR 2.6.1d [47]. Counts for each gene were obtained using featureCounts 1.5.1 [48].

The Gene Ontology (GO) term “inflammatory response” (GO:0006954) selected 860 putative inflammatory genes which were filtered to only those with at least two copies detected in at least 90 % of specimens. RNA read counts were normalized then transformed by centered log ratio (CLR).

#### 2.2.4 Integration of microbiota and host gene expression data

The *16S* ASVs were filtered and combined as described for the differential abundance analysis and also CLR-transformed. As the previous analysis showed minimal differences between in expression between the control arms from the two clinical sites [26], we again analyzed specimens from the entire trial as a single data set. We calculated the correlation between the gene counts and bacterial relative abundances, then filtered for r > 0.4 and Benjamani-Hochberg-adjusted p-value <0.05. The resulting genes were manually inspected for their most relevant GO annotation and grouped according to their immunological function and pro- or anti-inflammatory nature (Supplemental Table S1).

Normalized gene counts were used to perform random forest feature selection as implemented in the Boruta R package 7.0.0 [49]. Only the importance measures of statistically significant (p < 0.01) features were reported.

### 2.3 Quantification and statistical analyses

All statistical analyses were performed in R version 4. Alpha diversity comparisons were evaluated using the Wilcoxon rank sum test. Beta diversity was compared using Permutational Multivariate of Variance (PERMANOVA) using the adonis2 function of the vegan R package. The relationship between treatment arm and CST was assessed using multinomial logistic regression. RNAseq and 16S taxa correlations were calculated using Pearson coefficient. A significance threshold of α = 0.05 was used for the differential abundance hypothesis testing.

## 3 Results

### 3.1 Participant characteristics

The median age of the participants was 19 years (range: 13–24). All participants were assigned male sex at birth. No participants reported STI symptoms, and no physical exams revealed urethral discharge or other genital abnormality. None of the participants had clinical balanoposthitis or evidence of macroscopic inflammation. No GC infections were diagnosed, but NAAT for seven participants was positive for CT: five from Uganda and two from South Africa.

### 3.2 Microbiome 16S sequencing

**Fig. 1:**
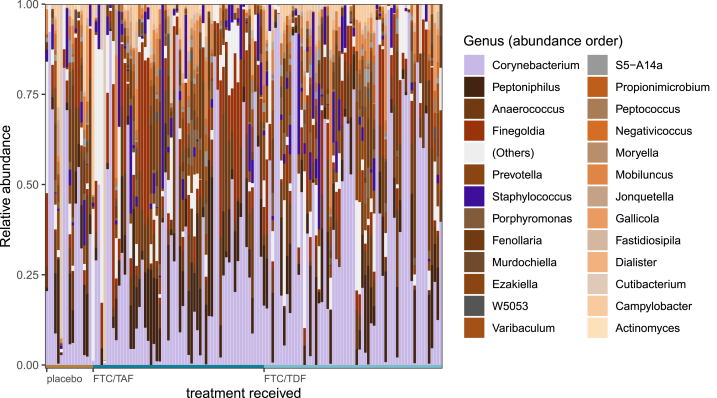
Top 25 genera. The top 25 most abundant bacterial genera across participants by study treatment received (anaerobic taxa in earth tones, aerobes in purples, uncultured or unidentified species in grays).

After filtering and contamination removal, 137 specimens from the 144 enrolled participants had sufficient bacterial DNA reads to proceed with analysis. The identified bacterial taxa include a variety of skin-associated Gram-positive and genital-associated anaerobic species in addition to Gram-negative enterics (Fig. 1). *Corynebacterium* was the most prevalent and abundant genus, appearing in 132 (97 %) of specimens at median relative abundance of 34 % (range: 0.14 %–98 %). Anaerobic species were highly abundant, including bacteria that are commonly found in bacterial vaginosis (an inflammatory dysbiosis of the vagina) including *Prevotella*, *Anaerococcus*, *Finegoldia* and *Porphyromonas*. There were no ASVs in the *Chlamydiaceae* family which includes *C. trachomatis*.

**Fig. 2:**
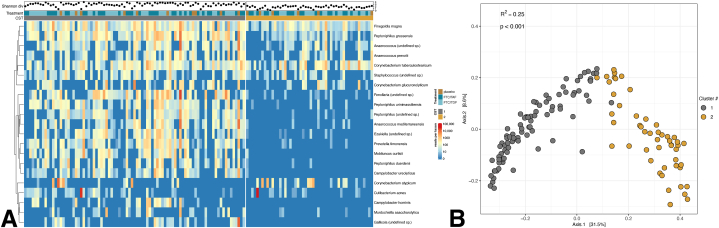
Clusters of bacterial taxa. (A) Heatmap of top 25 bacterial species by CST, (B) PCoA ordination of bacterial beta diversity by unweighted Unifrac distance, colored by CST.

Unsupervised partition around medioids clustering separated the unweighted Unifrac distances into two community structure types (CST) with distinct community structure (PERMANOVA R^2^ = 0.25 with p < 0.001) and alpha diversity (p = 2.39 × 10^−19^) (Fig. 2). Two clusters maximized the silhouette score with acceptable within sum of squares and gap statistics. CST1 was highly diverse with a median Shannon index of 3.05. CST2 was dominated by *Corynebacterium tuberculostearicum* and *Finegoldia magna* and, with median relative abundances of 21 % and 9.6 %, respectively. CST2 also had a significantly lower median Shannon index of 1.68 (Wilcoxon unpaired exact, p = 2.39 × 10^−19^). Though *F. magna* and *C. tuberculostearicum* were also abundant in CST1 (median relative abundances of 4.6 % and 2.5 %), they demonstrated similar relative abundance with *Anaerococcus, Campylobacter, Fenollaria, Finegoldia*, *Ezakiella*, *Mobiluncus*, and *Peptinophilis* species without a clear dominant taxon.

### 3.3 Microbiome differences by study site

**Fig. 3:**
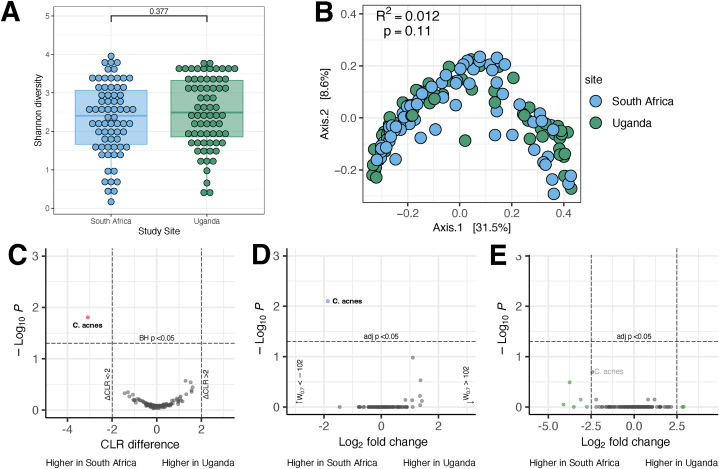
Comparisons by study site. Alpha (A) and beta (B) diversity by enrollment site; differential abundance testing with ALDEx2 (C), ANCOM-BC (D), and DESeq2 (E).

The 137 participants with 16S data included 69 (50.3 %) individuals from South Africa and 68 (49.7 %) from Uganda. We compared microbiota between the two study sites and found no differences in within-participant alpha diversity (Shannon entropy) or between-participant beta diversity (unweighted Unifrac distance) (Fig. 3A and B). Differential abundance testing identified *Cutibacterium acnes* as significantly higher in participants from South Africa compared to those from Uganda by ALDEx2 with a CLR difference of 3.3 between sites (Wilcoxon rank test with Benjamani-Hochberg correction p = 0.0184). ANCOM-BC identified the same ASV with a significant q-value (0.0079), but the log_2_fold change of 1.87 did not meet the recommended effect size threshold. DESeq2 did not identify any taxa as significantly differentially abundant. (Fig. 3, C-E).

### 3.4 Microbiome differences by parent study treatment arm

**Fig. 4:**
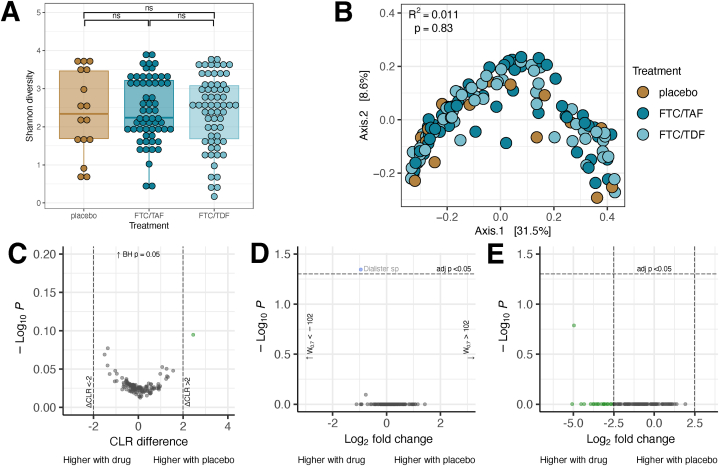
Comparisons by treatment arm. Alpha (A) and beta (B) diversity by drug received; differential abundance testing with ALDEx2 (C), ANCOM-BC (D), and DESeq2 (E).

We performed similar analyses for the bacterial populations in participants who received active drug versus placebo. The participants with *16S* data were equally distributed among treatment arms with FTC-TAF and FTC-TDF (n = 59 and 62, respectively, χ^2^ p = 0.437). All 16 participants who received placebo had sufficient sequences for analysis. We found no differences in alpha or beta diversity (Fig. 4A and B) between placebo and FTC-TAF or FTC-TDF regimens. Combining both treatment groups and comparing to placebo, no species were significantly differentially abundant by any of the three tools (Fig. 4, C-E). An un-annotated *Dialister* species was identified with statistically significantly higher abundance in participants who received active drug by ANCOM-BC but did not meet the effect size threshold at only 1.9-fold more abundant. The treatment arm was not a significant predictor of CST (p = 0.32 for placebo, p = 0.99 for FTC/TAF, p = 0.88 for FTC/TDF).

### 3.5 Inflammatory gene expression in foreskin tissue and bacterial taxa

**Fig. 5:**
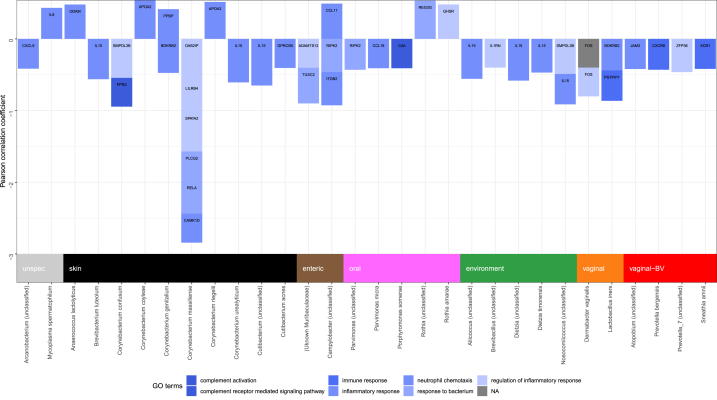
Bacterial taxa and inflammatory gene associations by immune function.

Expression of 40 inflammatory genes showed significant correlation with CLR-transformed relative abundance of 31 bacterial species (Table 1). Six genes had unknown inflammatory function and were therefore excluded. The remaining 34 genes were primarily pro-inflammatory with negative correlation with bacterial species abundance (Fig 5) without difference by usual environmental niche. IL-15 was the gene with the most correlations to microbial taxa, with significant negative correlations to seven bacterial taxa not typically associated with invasive infection: *Brevibacterium luteolum*, *Corynebacterium urealyticum*, *Dietzia timorensis*, and unannotated species in the *Cutibacterium, Corynebacterium, Dietzia,* and *Nosocomiicoccus* genera. Most of the bacterial taxa significantly correlated with other genes were gram-positive organisms which frequently colonize the skin. The CLR-transformed relative abundance of *Corynebacterium massilliense*, in particular, was associated with significantly lower expression of genes involved in regulation of inflammatory responses and neutrophil chemotaxis and activation. Anaerobes also found in the oral cavity such as *Parvimonas* and *Porphyromonas* were also correlated with primarily lower expression of inflammatory genes. However, the oral anaerobe *Rothia amarae* was associated with higher expression of the regulatory factor *GHSR* and an unclassified *Rothia* species was associated with higher expression of the pro-inflammatory gene *REG3G*. Species in three genera canonically associated with Bacterial Vaginosis (BV), *Atopobium, Prevotella,* and *Sneathia,* were correlated with lower expression of inflammatory genes, but one unannotated *Prevotella* ASV was negatively correlated with *ZFP36*, an immune regulatory gene.

**Table 1:** Bacterial taxa and human gene correlations.

Taxon (Genus species)	Gene Symbol	Pearson corr	adj p
(Uknown *Muribaculaceae* family)	ADAMTS12	−0.404	5.90E-04
(Uknown *Muribaculaceae* family)	TUSC2	−0.497	2.63E-06
*Aliicoccus* (unclassified sp)	IL15	−0.557	2.41E-08
*Anaerococcus lactolyticus*	ODAM	0.479	8.41E-06
*Arcanobacterium* (unclassified sp)	CXCL9	−0.415	3.31E-04
*Atopobium* (unclassified sp)	JAM3	−0.420	2.64E-04
*Brevibacillus* (unclassified sp)	IL1RN	−0.401	6.89E-04
*Brevibacterium luteolum*	IL15	−0.563	1.48E-08
*Campylobacter* (unclassified sp)	CCL11	0.492	3.68E-06
*Campylobacter* (unclassified sp)	ITGB2	−0.465	2.03E-05
*Campylobacter* (unclassified sp)	RIPK2	−0.461	2.55E-05
*Corynebacterium confusum*	FPR3	−0.402	6.46E-04
*Corynebacterium confusum*	MS4A2	−0.439	9.72E-05
*Corynebacterium confusum*	SMPDL3B	−0.543	7.86E-08
*Corynebacterium coyleae*	APOA2	0.542	8.40E-08
*Corynebacterium genitalium*	BDKRB2	−0.474	1.14E-05
*Corynebacterium genitalium*	POLB	−0.434	1.23E-04
*Corynebacterium genitalium*	PPBP	0.414	3.52E-04
*Corynebacterium massiliense*	CAMK1D	−0.405	5.56E-04
*Corynebacterium massiliense*	DAB2IP	−0.595	7.04E-10
*Corynebacterium massiliense*	KDM6B	−0.482	6.95E-06
*Corynebacterium massiliense*	LILRB4	−0.419	2.83E-04
*Corynebacterium massiliense*	PLCG2	−0.412	4.01E-04
*Corynebacterium massiliense*	PRCP	−0.469	1.51E-05
*Corynebacterium massiliense*	RELA	−0.454	3.88E-05
*Corynebacterium massiliense*	SPATA2	−0.556	2.43E-08
*Corynebacterium riegelii*	APOA2	0.516	6.69E-07
*Corynebacterium urealyticum*	IL15	−0.607	2.22E-10
*Cutibacterium* (unclassified sp)	IL15	−0.650	1.75E-12
*Cutibacterium* (unclassified sp)	S1PR3	−0.407	5.03E-04
*Cutibacterium* (unclassified sp)	TLR1	−0.402	6.55E-04
*Cutibacterium acnes*	GPRC5B	−0.405	5.46E-04
*Dermabacter vaginalis*	FOS	−0.401	6.84E-04
*Dietzia* (unclassified sp)	IL15	−0.583	2.48E-09
*Dietzia timorensis*	IL15	−0.470	1.49E-05
*Enhydrobacter* (unclassified sp)	HDAC4	−0.423	2.21E-04
*Lactobacillus iners*	BDKRB2	−0.440	8.68E-05
*Lactobacillus iners*	POLB	−0.428	1.72E-04
*Lactobacillus iners*	PSTPIP1	−0.423	2.21E-04
*Mycoplasma spermatophilum*	IL9	0.432	1.36E-04
*Nosocomiicoccus* (unclassified sp)	IL15	−0.424	2.11E-04
*Nosocomiicoccus* (unclassified sp)	SMPDL3B	−0.489	4.44E-06
*Parvimonas* (unclassified sp)	RIPK2	−0.430	1.44E-04
*Parvimonas micra*	CCL18	−0.409	4.56E-04
*Porphyromonas somerae*	C4A	−0.409	4.52E-04
*Prevotella bergensis*	CXCR6	−0.430	4.52E-04
*Prevotella_7* (unclassified sp)	ZFP36	−0.464	1.51E-04
*Rothia* (unclassified sp)	REG3G	0.534	2.18E-05
*Rothia amarae*	EGFR	−0.468	1.62E-07
*Rothia amarae*	GHSR	0.480	1.66E-05
*Sneathia amnii*	XCR1	−0.419	7.99E-06

We performed a similar analysis, grouping bacterial ASVs at the genus level (Supplemental Table S2). Eight genes showed correlation with nine bacterial genera. As expected, all the correlated genes and bacterial genera were also identified in the species-level analysis.

**Fig. 6:**
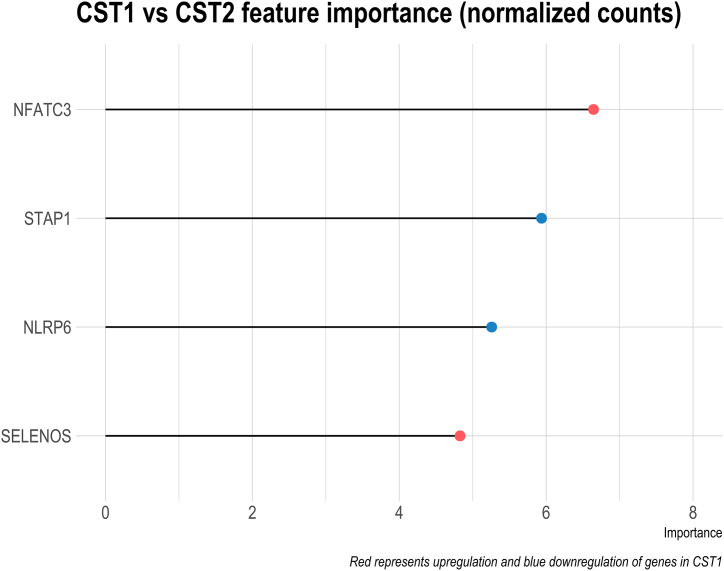
Random Forest Feature Importance. Comparing RNAseq results between CST 1 and CST 2.

We conducted a separate query of associations between inflammatory genes and the CSTs described in Fig 2A distinguished by high and low diversity bacterial populations. In the random forest classification, four genes showed statistically significant associations. Nuclear Factor of Activated T cells 3 (*NFATC3*) and Selenoprotein S (*SELENOS*) showed higher expression in the highly diverse CST1 relative to CST2, while Signal Transducing Adapter Family Member 1 (*STAP1*) and Nod-like Receptor Pyrin domain-containing 6 (*NLRP6*) showed lower expression (Fig 6).

## 4 Discussion

To our knowledge, this study is the first to analyze the tissue-level microbiome of the foreskin. Consistent with previous reports using penile swabs [[1], [2], [3], [4], [5], [6], [7], [8], [9], [10], [11], [12], [13], [14],^50^], the identified bacteria are predominated by taxa commonly colonizing the skin (chiefly *Corynebacteria* spp) [51,52] in addition to anaerobic bacteria such as *Prevotella, Dialister, Murdochiella, Peptoniphilus,* and *Negativicoccus*. These anaerobic species have been associated with increased inflammation and HIV acquisition in uncircumcised men [4] and with bacterial vaginosis in women [53]. In the foreskin, we describe two major CSTs, one significantly more diverse than the other. We did not identify bacterial species that were differentially abundant between participants receiving placebo compared to emtricitabine with either of the two forms of tenofovir. While vaginal-associated species such as *Lactobacillus* and *Gardnerella* take up tenofovir from their environment [19,20], further studies of more prolonged ARV use may better elucidate whether there is an interaction between ARVs and the bacterial or viral communities of the penis.

The overall composition of the foreskin bacterial community did not appear to differ by study site; however, we did find *Cutibacterium acnes* to be significantly more abundant in South African than Ugandan participants. *C. acnes* is typically resident in the deep dermis in association with sebaceous glands and hair follicles [54], which our study sampled by digesting full-thickness specimens rather than resuspending skin swabs. While its contribution to its namesake acne vulgaris is debated, it is otherwise non-pathogenic in immunocompetent hosts without artificial material [54]. It is frequently identified in surgical cultures, with unclear significance to sterility, likely due to its resistance to surgical sterilization techniques and transection of deep dermal structures during surgery [55]. The differential abundance we observed may have been caused by different surgical preparation techniques at the two sites or by an actual difference in bacterial populations. As there were no surgical complications observed during the study, the difference does not appear to have clinical significance.

Our inflammatory gene analysis primarily identified an inverse relationship between expression of genes associated with response to bacteria and skin commensals such as *Corynebacterium* and *Cutibacterium*, consistent with non-inflammatory commensal colonization. IL-15, the most commonly correlated gene, is a pleotropic cytokine secreted by a narrow range of cell types found in foreskin tissue including epithelial cells, fibroblasts, Langerhans cells, and macrophages [56]. It has broad immunostimulatory function, promoting NK cell differentiation and survival [57], inflammatory cytokine production by macrophages [58] and dendritic cells [59], neutrophil activation, survival, and phagocytosis [60], germinal center B cell proliferation [61], CD8^+^ T cell survival [62], and it is required for development of skin-resident memory CD8^+^ T cells [63]. Its lower expression correlating with higher relative abundances of commensal bacteria is consistent with dampened inflammatory signaling corresponding to prolonged exposure to commensal bacteria.

When examining gene expression between the participant groups with high versus low diversity bacterial communities, we did not confirm previous findings in penile swabs [4,6,12] and the vagina [64] that highly diverse, predominantly anaerobic communities are associated with increased inflammation. While we frequently identified anaerobic taxa, individual species found previously to be inflammatory were present across fewer specimens than in studies using surface swabs, possibly reducing our power to confirm prior associations. Another explanation could be methodological: rather than measuring secreted cytokines, we processed the entire tissue specimen for bulk RNAseq, which likely included many cells not directly interacting with bacteria or immune cells. Alternatively, the pre-procedure sterilization or the surgery itself may have preferentially removed inflammatory species. The sterilization or collection could also have altered host-bacterial interactions, though the rapid timeframe makes this explanation seem less likely. Lastly, the anaerobic strains in our cohort may employ different metabolic or virulence strategies that render them less inflammatory than those in other cohorts, however thus far no studies have investigated strain level differences in penile bacteria.

In the vagina, high diversity communities are inflammatory [64] and associated with increased risk of adverse outcomes including HIV acquisition [65,66] and preterm birth [67]. The foreskin microbiome had two obvious CSTs, one highly diverse CST1 and a less diverse CST2. We hypothesized that the more diverse CST1 would be associated with higher levels of genes related to inflammation, as in the vagina. However, when comparing gene expression between CST1 versus CST2 specimens, four genes were differentially expressed not including canonical innate antibacterial cytokine responses such as TNF-ɑ and IL-6. While named for its key activity in T-Cell Receptor (TCR) signaling, NFAT family members play a broad role in cell differentiation in the immune system and beyond [68], including in B cells [69], Toll-like receptor (TLR) signaling in monocytes [70], and proliferation in perivascular tissue [71] and keratinocytes [72]. Higher expression of NFATC3 in CST1 specimens may represent increased proliferation or signaling from these cells. SELENOS is up-regulated by cytokines such as IL-1β and TNF-α via NF-κB and in turns acts to suppress cytokine secretion in macrophages [73]. Its higher expression in CST1 samples is consistent with increased suppression of chronic inflammation. STAP1 has the best evidence for signaling downstream of the B-Cell Receptor (BCR) [74], and lower expression could represent a response to sustained signaling (although this was not supported by the expected changes in other genes). NLRP6 is both an inducer and a component of the inflammasome, binds directly to the lipopolysaccharide of gram negative or lipoteichoic acid of gram positive bacterial cell membranes, and plays both a pro- and anti-inflammatory role in tissues such as liver, kidney, and intestine [75]. Given these contrasting roles, its lower expression is difficult to interpret with the available data. None of the identified genes plays a direct role in regulating bacterial sensing or inflammatory response.

In summary, we report the first tissue-level examination of the bacterial microbiome of the foreskin, with no effect of brief antiretroviral drug exposure. Correlating the abundance of bacterial taxa with RNAseq data revealed largely negative correlations with genes involved in the inflammatory response, consistent with maintenance of immune tolerance of these commensal organisms.

## Ethics declarations

The CHAPS trial was registered at clinicaltrials.gov as NCT03986970 on 14/06/2019. Ethical clearance to conduct the trial was obtained from the South African Health Products Regulatory Authority (20181004), the Uganda Virus Research Institute research and ethics committee (GC/127/18/12/680), Uganda National Council of Science and Technology (HS 2534), Uganda National Drug Authority (618/NDA/DPS/09/2019), and the London School of Hygiene and Tropical Medicine research ethics committee (Ref:17403). All participants (or their proxies/legal guardians in the case of minors, who provided assent) provided informed consent to participate in the study. The Swedish Ethics Review Authority approved the transcriptome studies of the collected specimens at the Karolinska Institutet (2020-00941). The ethics approval for the microbiome analysis was granted by the Seattle Children's Institutional Review Board (STUDY00003430). The trial was conducted in accordance with South African Good Clinical Practice (SA-GCP), ICH76 GCP and ICMJE guidelines.

## Data availability statement

Raw nucleotide data have been deposited in the Short Read Archive and are publicly available under accession numbers PRJNA903228 (16S) and PRJNA884284 (RNASeq). R code to reproduce the analysis is available [76]. The completed STORMS (Strengthening The Organizing and Reporting of Microbiome Studies) checklist [77] has been completed for this project [78]. Any additional information required to analyze the data reported in this paper is available from the corresponding author upon request.

## CRediT authorship contribution statement

**Brandon S. Maust:** Writing – review & editing, Writing – original draft, Visualization, Project administration, Methodology, Investigation, Formal analysis, Data curation. **Stefan Petkov:** Writing – review & editing, Writing – original draft, Visualization, Validation, Software, Methodology, Investigation, Formal analysis, Data curation, Resources, Funding acquisition, Conceptualization. **Carolina Herrera:** Writing – review & editing, Conceptualization, Writing – review & editing, Data curation. **Colin Feng:** Writing – review & editing, Conceptualization, Methodology, Investigation. **Bryan P. Brown:** Writing – review & editing, Software, Methodology, Investigation. **Limakatso Lebina:** Writing – review & editing, Conceptualization. **Daniel Opoka:** Writing – review & editing, Conceptualization. **Andrew Ssemata:** Writing – review & editing, Conceptualization. **Natasha Pillay:** Writing – review & editing, Conceptualization. **Jennifer Serwanga:** Writing – review & editing, Conceptualization. **Portia Seatlholo:** Writing – review & editing, Conceptualization. **Patricia Namubiru:** Writing – review & editing, Conceptualization. **Geoffrey Odoch:** Writing – review & editing, Conceptualization. **Susan Mugaba:** Writing – review & editing, Conceptualization. **Thabiso Seiphetlo:** Writing – review & editing, Conceptualization. **Pontiano Kaleebu:** Writing – review & editing, Conceptualization. **Neil Martinson:** Writing – review & editing, Conceptualization. **Francesca Chiodi:** Writing – review & editing, Writing – original draft, Resources, Methodology, Investigation, Formal analysis, Conceptualization. **Julie Fox:** Writing – review & editing, Project administration, Conceptualization.

## Declaration of competing interest

The authors declare that they have no known competing financial interests or personal relationships that could have appeared to influence the work reported in this paper.

## References

[1] Nelson D.E., Dong Q., Van der Pol B., Toh E., Fan B., Katz B.P., Mi D., Rong R., Weinstock G.M., Sodergren E., Fortenberry J.D. (2012). Bacterial communities of the coronal sulcus and distal urethra of adolescent males. PLoS One.

[2] Carda-Diéguez M., Cárdenas N., Aparicio M., Beltrán D., Rodríguez J.M., Mira A. (2019). Variations in vaginal, penile, and oral microbiota after sexual intercourse: a case report. Front. Med..

[3] Onywera H., Williamson A.L., Ponomarenko J., Meiring T.L. (2020). The penile microbiota in uncircumcised and circumcised men: relationships with HIV and human papillomavirus infections and cervicovaginal microbiota. Front. Med..

[4] Prodger J.L., Abraham A.G., Tobian A.A., Park D.E., Aziz M., Roach K., Gray R.H., Buchanan L., Kigozi G., Galiwango R.M. (2021). Penile bacteria associated with HIV seroconversion, inflammation, and immune cells. JCI Insight.

[5] Liu C.M., Hungate B.A., Tobian A.A., Ravel J., Prodger J.L., Serwadda D., Kigozi G., Galiwango R.M., Nalugoda F., Keim P. (2015). Penile microbiota and female partner bacterial vaginosis in rakai, Uganda. mBio.

[6] Galiwango R.M., Park D.E., Huibner S., Onos A., Aziz M., Roach K., Anok A., Nnamutete J., Isabirye Y., Wasswa J.B. (2022). Immune milieu and microbiome of the distal urethra in Ugandan men: impact of penile circumcision and implications for HIV susceptibility. Microbiome.

[7] Li M., Mao J.X., Jiang H.H., Huang C.M., Gao X.H., Zhang L. (2021). Microbiome profile in patients with adult balanoposthitis: relationship with redundant prepuce, genital mucosa physical barrier status and inflammation. Acta Derm. Venereol..

[8] Mehta S.D., Zhao D., Green S.J., Agingu W., Otieno F., Bhaumik R., Bhaumik D., Bailey R.C. (2020). The microbiome composition of a man's penis predicts incident bacterial vaginosis in his female sex partner with high accuracy. Front. Cell. Infect. Microbiol..

[9] Mehta S.D., Nandi D., Agingu W., Green S.J., Bhaumik D.K., Bailey R.C., Otieno F. (2020). Vaginal and penile microbiome associations with HSV-2 in women and their male sex partners. J. Infect. Dis..

[10] Onywera H., Williamson A.L., Cozzuto L., Bonnin S., Mbulawa Z.Z.A., Coetzee D., Ponomarenko J., Meiring T.L. (2020). The penile microbiota of Black South African men: relationship with human papillomavirus and HIV infection. BMC Microbiol..

[11] Plummer E.L., Vodstrcil L.A., Danielewski J.A., Murray G.L., Fairley C.K., Garland S.M., Hocking J.S., Tabrizi S.N., Bradshaw C.S. (2018). Combined oral and topical antimicrobial therapy for male partners of women with bacterial vaginosis: acceptability, tolerability and impact on the genital microbiota of couples - a pilot study. PLoS One.

[12] Liu C.M., Prodger J.L., Tobian A.A.R., Abraham A.G., Kigozi G., Hungate B.A., Aziz M., Nalugoda F., Sariya S., Serwadda D. (2017). Penile anaerobic dysbiosis as a risk factor for HIV infection. mBio.

[13] Liu C.M., Hungate B.A., Tobian A.A., Serwadda D., Ravel J., Lester R., Kigozi G., Aziz M., Galiwango R.M., Nalugoda F. (2013). Male circumcision significantly reduces prevalence and load of genital anaerobic bacteria. mBio.

[14] Price L.B., Liu C.M., Johnson K.E., Aziz M., Lau M.K., Bowers J., Ravel J., Keim P.S., Serwadda D., Wawer M.J., Gray R.H. (2010). The effects of circumcision on the penis microbiome. PLoS One.

[15] Weller S., Davis K. (2002). Condom effectiveness in reducing heterosexual HIV transmission. Cochrane Database Syst. Rev..

[16] Silverman B.G., Gross T.P. (1997). Use and effectiveness of condoms during anal intercourse. A review. Sex. Transm. Dis..

[17] Spinner C.D., Boesecke C., Zink A., Jessen H., Stellbrink H.J., Rockstroh J.K., Esser S. (2016). HIV pre-exposure prophylaxis (PrEP): a review of current knowledge of oral systemic HIV PrEP in humans. Infection.

[18] Grulich A.E., Bavinton B.R. (2022). Scaling up preexposure prophylaxis to maximize HIV prevention impact. Curr. Opin. HIV AIDS.

[19] Taneva E., Sinclair S., Mesquita P.M., Weinrick B., Cameron S.A., Cheshenko N., Reagle K., Frank B., Srinivasan S., Fredricks D. (2018). Vaginal microbiome modulates topical antiretroviral drug pharmacokinetics. JCI Insight.

[20] Klatt N.R., Cheu R., Birse K., Zevin A.S., Perner M., Noël-Romas L., Grobler A., Westmacott G., Xie I.Y., Butler J. (2017). Vaginal bacteria modify HIV tenofovir microbicide efficacy in African women. Science.

[21] Sutton T.D.S., Hill C. (2019). Gut bacteriophage: current understanding and challenges. Front. Endocrinol..

[22] Fulcher J.A., Li F., Cook R.R., Zabih S., Louie A., Okochi H., Tobin N.H., Gandhi M., Shoptaw S., Gorbach P.M., Aldrovandi G.M. (2019). Rectal microbiome alterations associated with oral human immunodeficiency Virus pre-exposure prophylaxis. Open Forum Infect. Dis..

[23] Dubé M.P., Park S.Y., Ross H., Love T.M.T., Morris S.R., Lee H.Y. (2018). Daily HIV pre-exposure prophylaxis (PrEP) with tenofovir disoproxil fumarate-emtricitabine reduced Streptococcus and increased Erysipelotrichaceae in rectal microbiota. Sci. Rep..

[24] Perler B.K., Reinhart E.M., Montgomery M., Maynard M., Shapiro J.M., Belenky P., Chan P.A. (2021). Evaluation of the microbiome in men taking pre-exposure prophylaxis for HIV prevention. AIDS Behav..

[25] Hughes S.M., Levy C.N., Calienes F.L., Stekler J.D., Pandey U., Vojtech L., Berard A.R., Birse K., Noël-Romas L., Richardson B. (2020). Treatment with commonly used antiretroviral drugs induces a type I/III interferon signature in the gut in the absence of HIV infection. Cell Rep Med.

[26] Petkov S., Herrera C., Else L., Lebina L., Opoka D., Seiphetlo T.B., Pillay A.A., Mugaba S., Namubiru P., Odoch G. (2022). Short-term oral pre-exposure prophylaxis against HIV-1 modulates the transcriptome of foreskin tissue in young men in Africa. Front. Immunol..

[27] Rametse C.L., Webb E.L., Herrera C., Alinde B., Besethi A., Motaung B., Mbangiwa T., Leach L., Sebaa S., Pillay A.A.P. (2023). A randomized clinical trial of on-demand oral pre-exposure prophylaxis does not modulate lymphoid/myeloid HIV target cell density in the foreskin. AIDS.

[28] Nash S., Dietrich J., Ssemata A.S., Herrera C., O'Hagan K., Else L., Chiodi F., Kelly C., Shattock R., Chirenje M. (2020). Combined HIV Adolescent Prevention Study (CHAPS): comparison of HIV pre-exposure prophylaxis regimens for adolescents in sub-Saharan Africa-study protocol for a mixed-methods study including a randomised controlled trial. Trials.

[29] Maust, B.S. Foreskin Tissue DNA Extraction protocol. 10.17504/protocols.io.4r3l2774jg1y/v1..

[30] Dabee S., Mugo N., Mudhune V., McLellan-Lemal E., Peacock S., O'Connor S., Njoroge B., Nyagol B., Thurman A.R., Ouma E. (2022). Genital microbiota of women using a 90 day tenofovir or tenofovir and levonorgestrel intravaginal ring in a placebo controlled randomized safety trial in Kenya. Sci. Rep..

[31] Martin M. (2011). Cutadapt removes adapter sequences from high-throughput sequencing reads. EMBnet.journal.

[32] Callahan B.J., McMurdie P.J., Rosen M.J., Han A.W., Johnson A.J., Holmes S.P. (2016). DADA2: high-resolution sample inference from Illumina amplicon data. Nat. Methods.

[33] Quast C., Pruesse E., Yilmaz P., Gerken J., Schweer T., Yarza P., Peplies J., Glöckner F. (2013). The SILVA ribosomal RNA gene database project: improved data processing and web-based tools. Nucleic acids research.

[34] Brown B.P. (2021).

[35] McMurdie P.J., Holmes S. (2013). phyloseq: an R package for reproducible interactive analysis and graphics of microbiome census data. PLoS One.

[36] Oksanen J., Blanchet F.G., Friendly M., Kindt R., Legendre P., McGlinn D., Minchin P.R., O'Hara R.B., Simpson G.L., Solymos P. (2020).

[37] Nawrocki E.P. (2009).

[38] Guindon S., Dufayard J.F., Lefort V., Anisimova M., Hordijk W., Gascuel O. (2010). New algorithms and methods to estimate maximum-likelihood phylogenies: assessing the performance of PhyML 3.0. Syst. Biol..

[39] Davis N., Proctor D., Holmes S., Relman D., Callahan B. (2018). Simple statistical identification and removal of contaminant sequences in marker-gene and metagenomics data. Microbiome.

[40] Brown, B.B. microfiltR..

[41] Fernandes A.D., Reid J.N., Macklaim J.M., McMurrough T.A., Edgell D.R., Gloor G.B. (2014). Unifying the analysis of high-throughput sequencing datasets: characterizing RNA-seq, 16S rRNA gene sequencing and selective growth experiments by compositional data analysis. Microbiome.

[42] Lin H., Peddada S.D. (2020). Analysis of compositions of microbiomes with bias correction. Nat. Commun..

[43] Love M.I., Huber W., Anders S. (2014). Moderated estimation of fold change and dispersion for RNA-seq data with DESeq2. Genome Biol..

[44] Nearing J.T., Douglas G.M., Hayes M.G., MacDonald J., Desai D.K., Allward N., Jones C.M.A., Wright R.J., Dhanani A.S., Comeau A.M., Langille M.G.I. (2022). Microbiome differential abundance methods produce different results across 38 datasets. Nat. Commun..

[45] Lin H., Peddada S.D. (2020). Analysis of microbial compositions: a review of normalization and differential abundance analysis. NPJ Biofilms Microbiomes.

[46] Ewels P., Magnusson M., Lundin S., Kaller M. (2016). MultiQC: summarize analysis results for multiple tools and samples in a single report. Bioinformatics.

[47] Dobin A., Davis C.A., Schlesinger F., Drenkow J., Zaleski C., Jha S., Batut P., Chaisson M., Gingeras T.R. (2013). STAR: ultrafast universal RNA-seq aligner. Bioinformatics.

[48] Liao Y., Smyth G.K., Shi W. (2014). featureCounts: an efficient general purpose program for assigning sequence reads to genomic features. Bioinformatics.

[49] Kursa M.B., Rudnicki W.R. (2010). Feature selection with the Boruta package. J Stat Softw.

[50] Srinivasan S., Chambers L.C., Tapia K.A., Hoffman N.G., Munch M.M., Morgan J.L., Domogala D., Sylvan Lowens M., Proll S., Huang M.L. (2021). Urethral microbiota in men: association of Haemophilus influenzae and mycoplasma penetrans with nongonococcal urethritis. Clin. Infect. Dis..

[51] Human Microbiome Project C. (2012). Structure, function and diversity of the healthy human microbiome. Nature.

[52] Oh J., Byrd A.L., Park M., Program N.C.S., Kong H.H., Segre J.A. (2016). Temporal stability of the human skin microbiome. Cell.

[53] Dabee S., Passmore J.S., Heffron R., Jaspan H.B. (2021). The complex link between the female genital microbiota, genital infections, and inflammation. Infect. Immun..

[54] Achermann Y., Goldstein E.J., Coenye T., Shirtliff M.E. (2014). Propionibacterium acnes: from commensal to opportunistic biofilm-associated implant pathogen. Clin. Microbiol. Rev..

[55] Lee M.J., Pottinger P.S., Butler-Wu S., Bumgarner R.E., Russ S.M., Matsen F.A. (2014). Propionibacterium persists in the skin despite standard surgical preparation. J Bone Joint Surg Am.

[56] Perera P.Y., Lichy J.H., Waldmann T.A., Perera L.P. (2012). The role of interleukin-15 in inflammation and immune responses to infection: implications for its therapeutic use. Microb. Infect..

[57] Waldmann T.A., Tagaya Y. (1999). The multifaceted regulation of interleukin-15 expression and the role of this cytokine in NK cell differentiation and host response to intracellular pathogens. Annu. Rev. Immunol..

[58] Alleva D.G., Kaser S.B., Monroy M.A., Fenton M.J., Beller D.I. (1997). IL-15 functions as a potent autocrine regulator of macrophage proinflammatory cytokine production: evidence for differential receptor subunit utilization associated with stimulation or inhibition. J. Immunol..

[59] Mattei F., Schiavoni G., Belardelli F., Tough D.F. (2001). IL-15 is expressed by dendritic cells in response to type I IFN, double-stranded RNA, or lipopolysaccharide and promotes dendritic cell activation. J. Immunol..

[60] Girard D., Paquet M.E., Paquin R., Beaulieu A.D. (1996). Differential effects of interleukin-15 (IL-15) and IL-2 on human neutrophils: modulation of phagocytosis, cytoskeleton rearrangement, gene expression, and apoptosis by IL-15. Blood.

[61] Park C.S., Yoon S.O., Armitage R.J., Choi Y.S. (2004). Follicular dendritic cells produce IL-15 that enhances germinal center B cell proliferation in membrane-bound form. J. Immunol..

[62] Wu T.S., Lee J.M., Lai Y.G., Hsu J.C., Tsai C.Y., Lee Y.H., Liao N.S. (2002). Reduced expression of Bcl-2 in CD8+ T cells deficient in the IL-15 receptor alpha-chain. J. Immunol..

[63] Mackay L.K., Rahimpour A., Ma J.Z., Collins N., Stock A.T., Hafon M.L., Vega-Ramos J., Lauzurica P., Mueller S.N., Stefanovic T. (2013). The developmental pathway for CD103(+)CD8+ tissue-resident memory T cells of skin. Nat. Immunol..

[64] Anahtar M.N., Byrne E.H., Doherty K.E., Bowman B.A., Yamamoto H.S., Soumillon M., Padavattan N., Ismail N., Moodley A., Sabatini M.E. (2015). Cervicovaginal bacteria are a major modulator of host inflammatory responses in the female genital tract. Immunity.

[65] McClelland R.S., Lingappa J.R., Srinivasan S., Kinuthia J., John-Stewart G.C., Jaoko W., Richardson B.A., Yuhas K., Fiedler T.L., Mandaliya K.N. (2018). Evaluation of the association between the concentrations of key vaginal bacteria and the increased risk of HIV acquisition in African women from five cohorts: a nested case-control study. Lancet Infect. Dis..

[66] Masson L., Passmore J.A., Liebenberg L.J., Werner L., Baxter C., Arnold K.B., Williamson C., Little F., Mansoor L.E., Naranbhai V. (2015). Genital inflammation and the risk of HIV acquisition in women. Clin. Infect. Dis..

[67] Wylie K.M., Wylie T.N., Cahill A.G., Macones G.A., Tuuli M.G., Stout M.J. (2018). The vaginal eukaryotic DNA virome and preterm birth. Am. J. Obstet. Gynecol..

[68] Horsley V., Pavlath G.K. (2002). NFAT: ubiquitous regulator of cell differentiation and adaptation. J. Cell Biol..

[69] Venkataraman L., Francis D.A., Wang Z., Liu J., Rothstein T.L., Sen R. (1994). Cyclosporin-A sensitive induction of NF-AT in murine B cells. Immunity.

[70] Minematsu H., Shin M.J., Celil Aydemir A.B., Kim K.O., Nizami S.A., Chung G.J., Lee F.Y. (2011). Nuclear presence of nuclear factor of activated T cells (NFAT) c3 and c4 is required for Toll-like receptor-activated innate inflammatory response of monocytes/macrophages. Cell. Signal..

[71] Graef I.A., Chen F., Chen L., Kuo A., Crabtree G.R. (2001). Signals transduced by Ca(2+)/calcineurin and NFATc3/c4 pattern the developing vasculature. Cell.

[72] Santini M.P., Talora C., Seki T., Bolgan L., Dotto G.P. (2001). Cross talk among calcineurin, Sp1/Sp3, and NFAT in control of p21(WAF1/CIP1) expression in keratinocyte differentiation. Proc. Natl. Acad. Sci. U.S.A..

[73] Curran J.E., Jowett J.B., Elliott K.S., Gao Y., Gluschenko K., Wang J., Abel Azim D.M., Cai G., Mahaney M.C., Comuzzie A.G. (2005). Genetic variation in selenoprotein S influences inflammatory response. Nat. Genet..

[74] Ohya K., Kajigaya S., Kitanaka A., Yoshida K., Miyazato A., Yamashita Y., Yamanaka T., Ikeda U., Shimada K., Ozawa K., Mano H. (1999). Molecular cloning of a docking protein, BRDG1, that acts downstream of the Tec tyrosine kinase. Proc. Natl. Acad. Sci. U.S.A..

[75] Angosto-Bazarra D., Molina-Lopez C., Pelegrin P. (2022). Physiological and pathophysiological functions of NLRP6: pro- and anti-inflammatory roles. Commun. Biol..

[76] Maust B.S. GitHub repository. https://github.com/bmaust/CHAPS/.

[77] Mirzayi C., Renson A., Genomic Standards C., Massive A., Quality Control S., Zohra F., Elsafoury S., Geistlinger L., Kasselman L.J., Eckenrode K. (2021). Reporting guidelines for human microbiome research: the STORMS checklist. Nat. Med..

[78] Maust, B.S. manuscript STORMS checklist. 10.5281/zenodo.7312059..

